# Subjective and objective changes in visual quality after implantable collamer lens implantation for myopia

**DOI:** 10.3389/fmed.2025.1543864

**Published:** 2025-03-07

**Authors:** Li-li Nie, Xiang Ma, Ying Pei

**Affiliations:** Department of Ophthalmology, The Second Hospital of Jilin University, Jilin, China

**Keywords:** implantable collamer lens, objective visual quality, subjective visual quality, corneal refractive surgery, pupil size

## Abstract

With the wide application of implantable collamer lens (ICL) surgery for myopia correction, the range of refractive correction has expanded (up to −18.00 D for myopia), and the safety, effectiveness, predictability and stability of ICLs have been well documented. However, achieving good visual quality after ICL implantation has also become very important. This article systematically reviews objective and subjective visual quality after ICL surgery. First, parameters used to assess objective visual quality after ICL surgery are introduced, including higher-order aberrations, the modulation transfer function (MTF) cutoff (cycles per degree [cpd]), the Strehl 2D ratio (SR), and the objective scatter index (OSI). Notably, various post-operative objective visual quality measurements have been improving over time. However, halos and glare caused by ICL implantation are notable postoperative complications. In further discussions, we also focus on factors that can affect visual quality, such as ICL position changes, pupil size, and the ICL optical zone. Furthermore, measures to improve postoperative visual quality, such as the selection of the surgical incision and mode, are provided. This review explores the potential mechanisms, emphasizes the importance of pre- and postoperative measures, and provides guidance for good postoperative visual quality. Additionally, this review aims to address the factors influencing visual quality and postoperative outcomes to optimize vision after ICL implantation.

## 1 Introduction

Myopia, a common refractive error, has become a significant public health problem worldwide. It is estimated that by 2050, 50% of the world’s population will be myopic, and 10% will be highly myopic (≤ −6.00 D), which places a heavy economic burden on society as a whole (1). Over the past decade, refractive surgery has moved beyond traditional laser surgery. Since 2005, the U.S. Food and Drug Administration has approved the use of the implantable collamer lens (ICL^™^; STAAR Surgical, Nidau, Switzerland) as a supplement to increase the effect of the natural lens and achieve a wider range of refractive correction (up to −18.00 D for myopia).

The influence of different types of refractive surgery on postoperative visual quality has become a hot topic in ophthalmology. During the procedure, the ICL is inserted between the eye’s natural lens and the iris. The ICL works with the natural lens to refract light onto the retina, improving visual clarity without relying on glasses or contact lenses and without altering the structure of the eye. Additionally, the procedure is reversible, as the ICL can be removed if necessary (2). However, good visual acuity cannot fully reflect the visual function of the human eye, and some patients experience visual quality degradation, such as glare, blurred vision at night, monocular diplopia, a star-shaped change in vision and discomfort in near vision after surgery. Visual quality reflects not only the clinical effectiveness after surgery to a certain extent but also the subjective feelings of surgical patients, such as their visual comfort level. With increasing demand for better eye health, visual quality, and overall quality of life, whether ICLs can provide good optical quality after surgery is also worth studying. As with any surgical intervention, ICL surgery also has inherent risks and potential complications (Table 1). To date, many studies have compared the effects of ICLs and corneal refractive surgery on the basis of visual quality, corneal refractive power and quality of life. The purpose of this review is to summarize the data on postoperative visual quality after ICL surgery for the correction of myopia and myopic astigmatism, as well as to outline the control factors affecting visual quality and measures to improve vision after surgery.

**Table 1:** Complications of ICL implantation surgery.

Intraoperative complications	Conjunctival or intraocular hemorrhage
	Corneal epithelial defects, corneal edema
	Traumatic cataract
Postoperative complications	Corneal endothelial cell loss, corneal decompensation
	Cataract development, increased intraocular pressure
	The possibility of undercorrection or overcorrection
	Abnormal vault, ICL dislocation, ICL subluxation
	Anterior chamber pigment dispersion, iritis
	Retinal detachment, retinal tear, preretinal membrane formation

## 2 Visual quality after ICL implantation

### 2.1 Objective visual quality

In addition to the standard parameters of refractive surgery, such as postoperative intraocular pressure (IOP), anterior chamber depth (ACD), the optical quality of eyes implanted with ICLs has been evaluated *in vivo* to understand their optical performance. Optical quality has been evaluated in terms of ocular higher-order aberrations, retinal imaging quality, and intraocular scatter (3–9). Corneal higher-order aberrations (HOAs) are complex optical aberrations caused by the non-ideal aspherical surface of the cornea and cannot be corrected by glasses. HOAs have a significant effect on the quality of human vision, as they cause glare, halos, and loss of night vision. Higher-order aberrations also include spherical aberrations, coma aberrations, and trefoil aberrations (9, 10). Several studies have shown significant increases in the number of HOAs, including the RMSs of spherical, comet, clover, and higher-order aberrations, within 1 week after ICL implantation, which decrease at 3 months after surgery to a level similar to that noted before surgery (7, 11, 12). HOAs may be induced by the corneal incision (size and location), lens optics (higher magnification or higher spherical aberration) (13), or lens location (centration and/or tilt) (14) at the time of ICL implantation (11). The tilt of the ICL may be related to an increase in non-rotational symmetric aberrations, namely, coma, whereas the change in spherical aberration may be related to an increase in the negative spherical aberration of one’s own lens when the magnification of the ICL is increased. A significant increase in the number of HOAs after ICL may also be caused by the procedure itself or the inherent optical properties of the lens. Kayhan et al. reported that the number of corneal aberrations did not change after ICL implantation, whereas the number of internal aberrations and total HOAs (tHOAs) significantly changed (15). Similarly, Chen et al. (16) reported that the numbers of coma and spherical aberrations increased after ICL implantation. Ping-hui Wei et al. also proposed that the significant difference in the number of internal aberrations is due to the design of the ICL and its effect on the passage of light (12). Moreover, the number of ocular HOAs increases with increasing pupil diameter (17–19).

However, the potential advantages of ICL implantation over corneal refractive laser surgery (LASIK, SMILE, etc.) include higher contrast sensitivity (8, 20–22), higher magnification of retinal images, and fewer HOAs (3, 8, 23, 24). Luo et al. reported that the incidence of spherical and coma aberrations in SMILE-treated eyes was significantly greater than that in ICL-treated eyes and that the incidence of vertical and horizontal coma aberrations after ICL implantation was significantly lower than that of horizontal coma aberrations after SMILE. In addition, clover aberrations were more often observed after ICL compared with SMILE. Zheng et al. also analyzed the reasons why optical and visual qualities, such as tear film instability, corneal flaps, laser ablation, inflammatory stimulation, and edge effects, were better after ICL implantation compared with LASIK surgery (6). In addition, because the optical zone of larger pupils is easier to cover, patients with large dark pupils are recommended for ICL implantation to avoid visual quality problems caused by LASIK and spherical aberrations.

A double-pass optical quality analysis system (OQAS II; Visiometrics, Terrassa, Spain) was used to objectively measure retinal image quality and intraocular scatter, as the system is known for good repeatability and reliability. Three objective parameters were recorded: the modulation transfer function cutoff (MTF cutoff, cycles per degree [cpd]), the Strehl2D ratio (SR), and the objective scatter index (OSI) (25). The first two parameters are retinal image quality parameters, with higher values indicating higher optical quality. The OSI quantifies intraocular scattering, with lower values indicating lower intraocular scattering and better optical quality. The MTF cutoff is the frequency at which the MTF reaches a value of 0.01, i.e., the frequency at which the eye is able to focus an object on the retina with a contrast of 1%. The SR is the ratio of the central maximum of the point spread function (PSF) illuminance in the eye with aberrations to the central maximum expected in the corresponding aberration-free system; values range between 0 and 1, with an SR of 1 indicating a perfect, aberration-free system (26). Qin Qin et al. reported that the MTF and SR at 1 and 3 months after ICL implantation were increased compared with the preoperative values (7). Compared with those after corneal refractive surgery, the MTF cutoff values and Strehl2D ratios after SMILE and ICL implantation were not significantly different, suggesting that both procedures provide long-term good visual quality (3, 27). However, in terms of the MTF cutoff values, the evidence is contradictory. Qin et al. (28) reported higher postoperative MTF cutoff values in ICL-treated eyes than in SMILE-treated eyes, whereas Niu et al. (9) reported no significant difference between the two treatments. Scattering is another important factor affecting optical quality. The OSI was calculated as the ratio between the amount of light outside the dual-pass retinal intensity PSF image in the peripheral region (circles between 12 and 20 arc minutes) and the central region of the retinal image (circles with a radius of 1 arc minute). The OSI of the normal eye is approximately 1, whereas values above 5 indicate a highly dispersed system. However, Qin et al. reported that an ICL does not result in increased intraocular scattering because the thickness of the EVO-ICL ring is 100 to 200 microns, the thickness of the optical zone is only 40 to 50 microns, and the ICL is located in the ciliary sulcus with minimal tilt or displacement (28). Zhan et al. reported no statistically significant differences in the OSI or SR at 1, 3, or 6 months after ICL implantation (5). Moreover, Luo et al. reported no significant differences in the OSI or SR before and after surgery (24). In addition, age and preoperative SE are correlated with the postoperative OSI (25). Similarly, the optical quality parameters improved after ICL implantation. These positive results, including contrast sensitivity and optical quality, were also confirmed under different lighting conditions, namely, dark or glare conditions (29, 30).

### 2.2 Subjective visual quality

**Figure 1:**
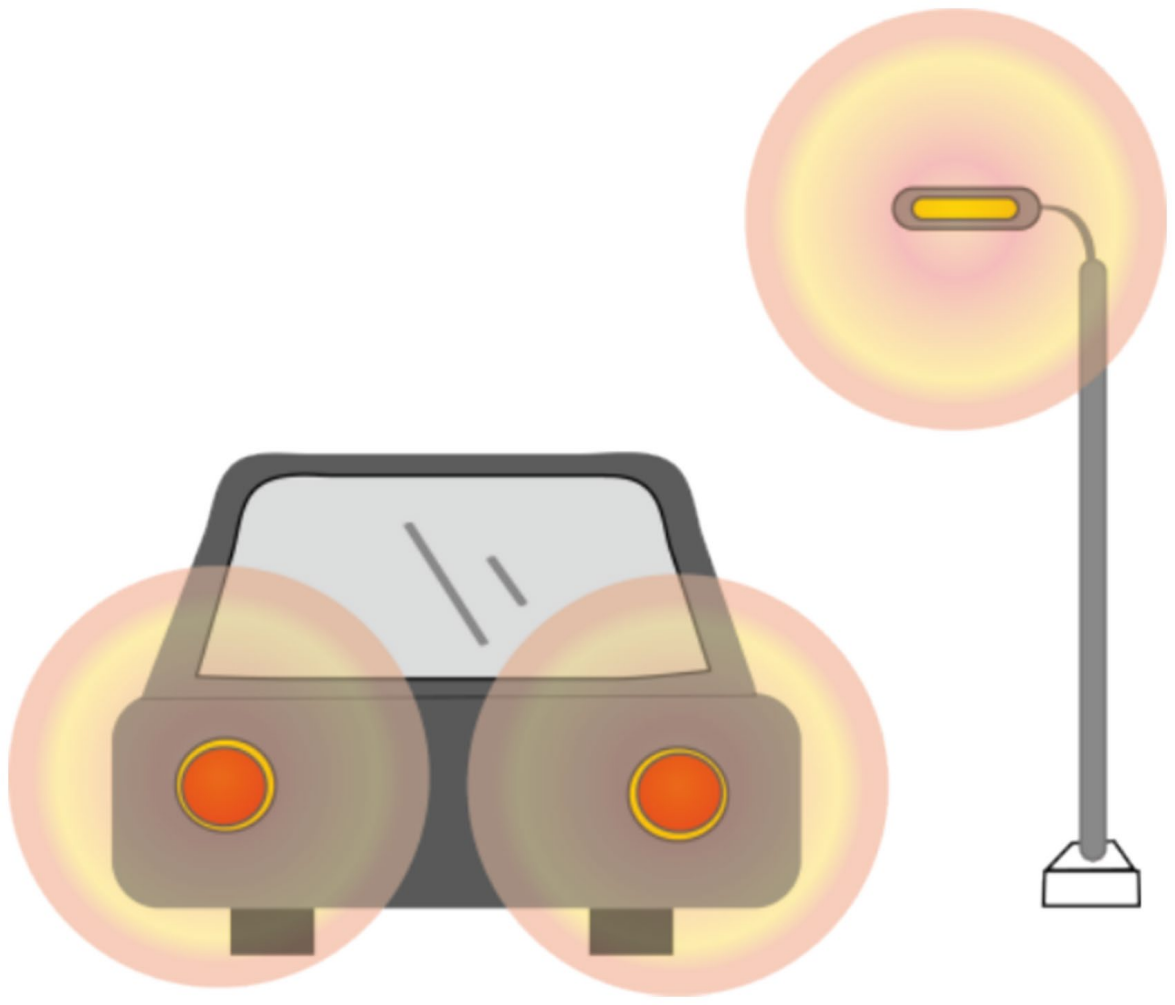
Halo: A bright colored ring around a light source.

**Figure 2:**
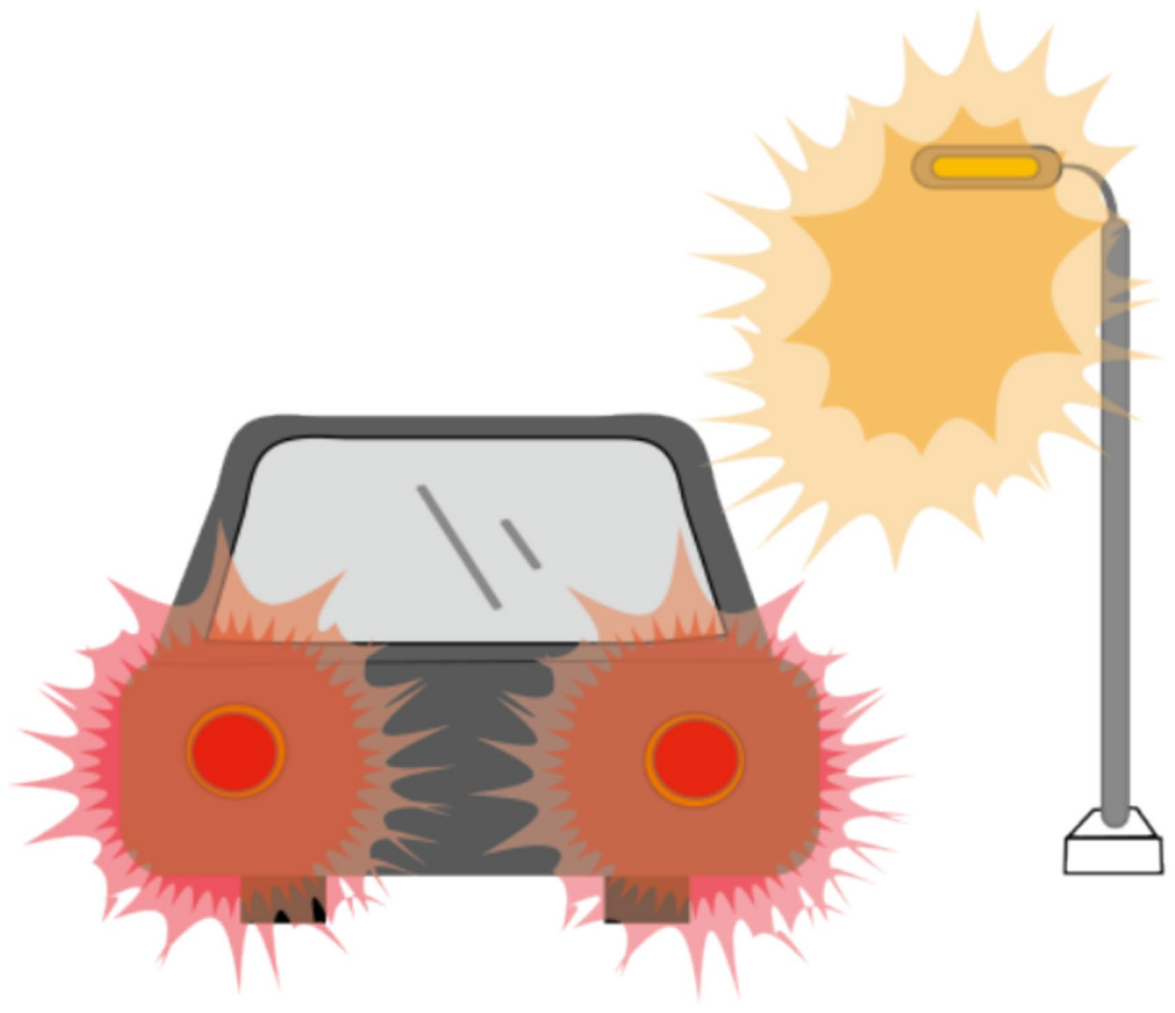
Glare: A bright spot in the center of a light source.

The objective visual quality after ICL implantation, as described previously, has been extensively studied. However, the subjective visual quality after ICL implantation has also been studied to some extent. In most studies, researchers have used McAlinden’s Quality of Vision (QoV) questionnaire (31) and the NEI-RQL-42 questionnaire (32) to investigate subjective visual parameters. More than 90% of patients experienced halos to some extent (Figure 1), and more than half experienced glare (Figure 2). Halos are described as glowing foggy rings surrounding a light source, whereas glare is defined as a contrast-reducing effect of stray light in a visual scene. However, most patients report that halos and glare only slightly disturb their vision or not at all. Notably, Eom et al. (33) reported that the mean durations of glare and halos after the implantation of a V4c ICL were 3.0 ± 3.4 and 3.1 ± 3.6 months, respectively. Liu et al. (34) reported that halos were no longer visible 3 months after ICL implantation. It has also been reported that a halo is the most common long-term visual disturbance after ICL implantation (35). Siedlecki et al. (36) reported an 80% incidence of halos and 60% for glare 2 years after surgery.

**Figure 3:**
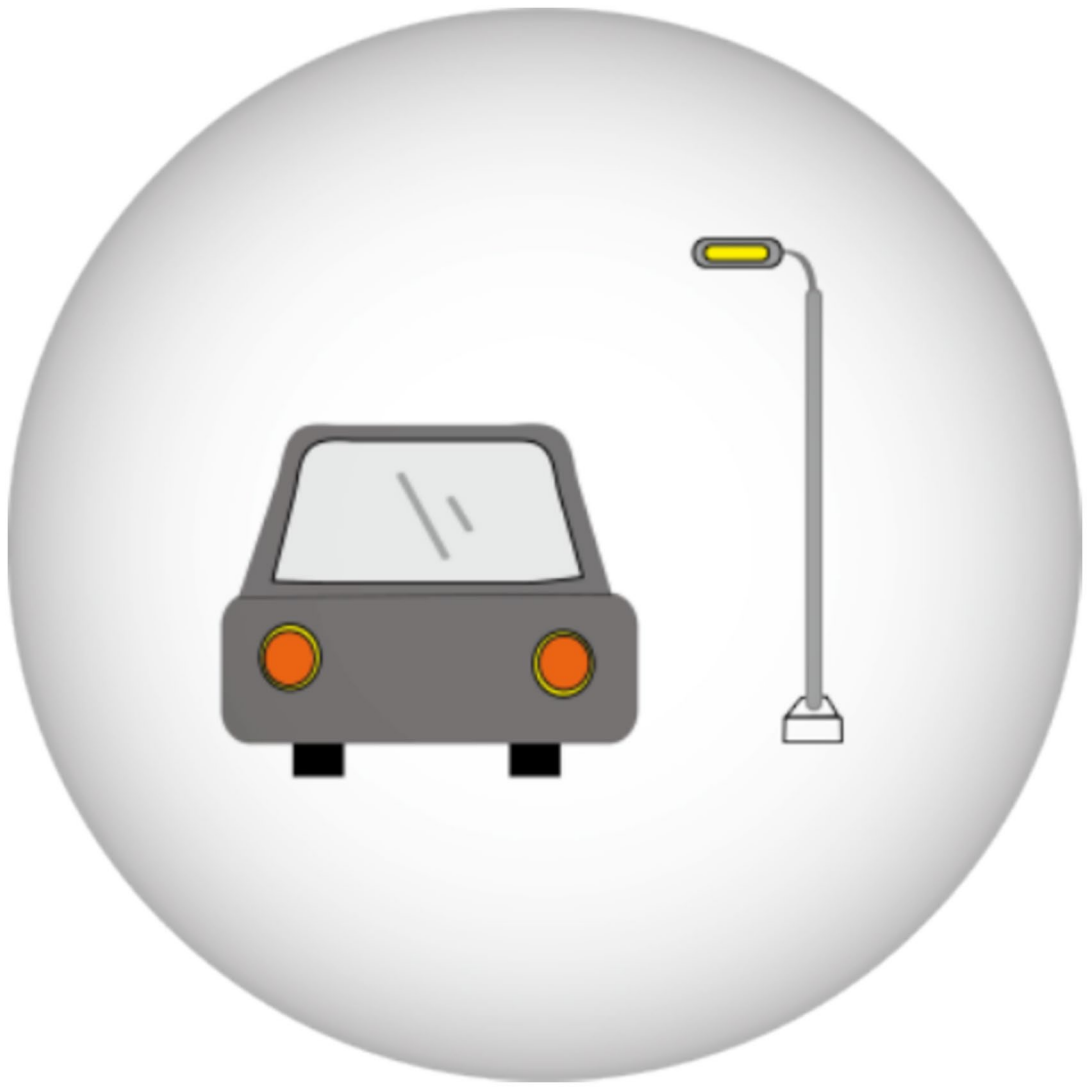
Ring-shaped dysphotopsia: A relatively large, sharp circular halo around a bright light source.

Recent studies revealed that annular visual impairment has become the third most frequently reported visual symptom in questionnaires (33, 37). Ring-shaped dysphotopsia differs from a halo that occurs after ICL implantation with a central hole and may appear alone or in combination with a halo (Figure 3). The former can be described as a relatively large and sharp circular halo around a bright light source, whereas the latter is a ring with significantly less glare. Eppig et al. and Eom et al. reported that ring-shaped dysphotopsia may be directly related to the presence of the central hole in the ICL and is a specific visual sequelae caused by the implantation of a V4c ICL (33, 37). However, annular visual impairment is not thought to be related to the central foramen, as it has also been reported to occur after the implantation of an ICL without a central hole (2). Circumferential visual disturbance was initially high at 1 week after surgery and gradually decreased to a lower level at 1, 3, and 6 months after surgery, with a mean time to complete cessation of 2.9 ± 3.8 months (29).

In addition, Martinez-Plata et al. (29) reported no effect of the preoperative ICL power or pupil diameter on the ring-shaped dysphotopsia subscale.

## 3 Factors affecting the quality of vision

### 3.1 Position change of the ICL

**Figure 4:**
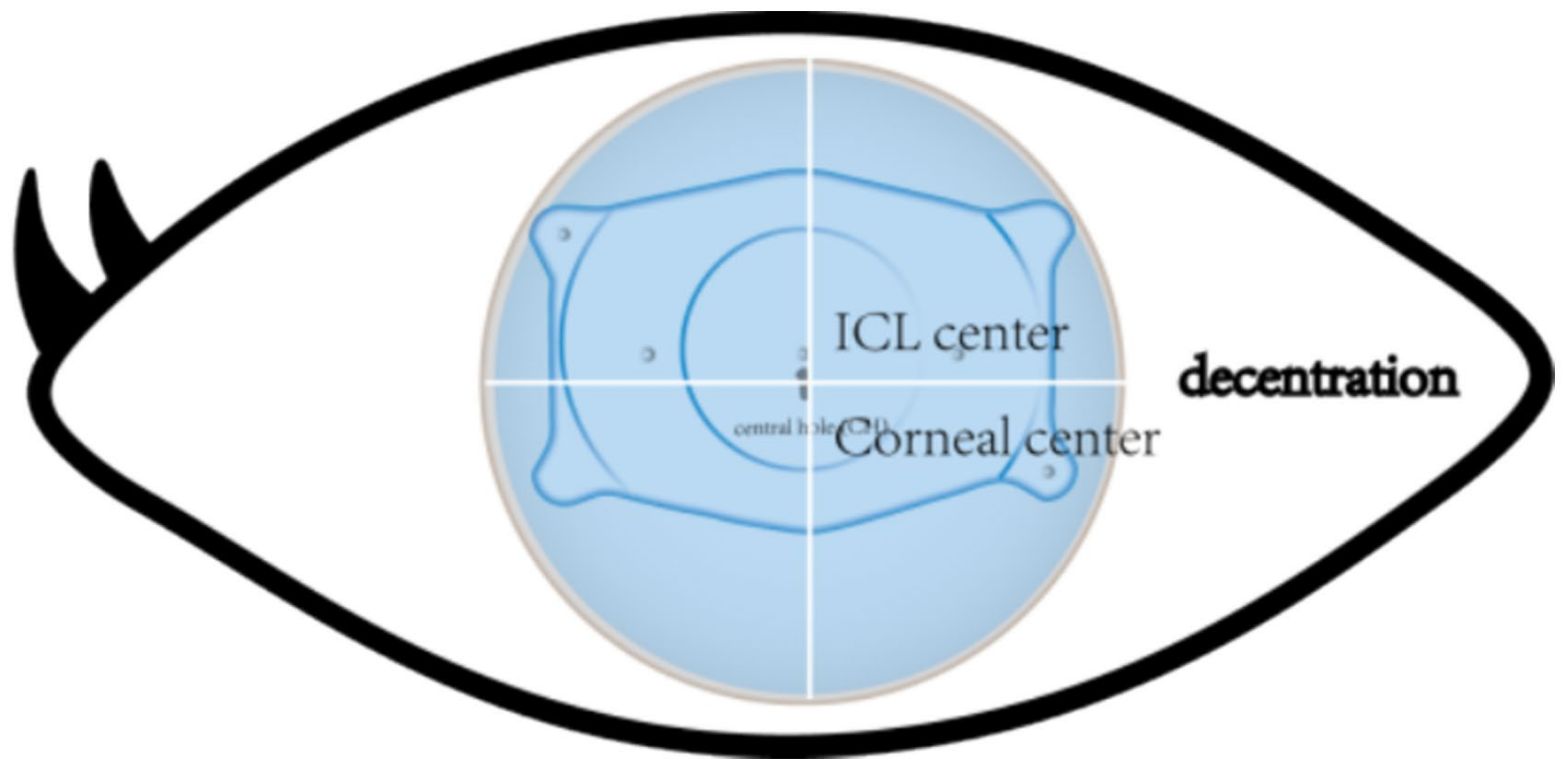
The point of the cross line of the upper and lower cornea is the corneal center. The arrows indicate the central hole of the ICL. The central hole shown is not in the same position as the central point of the cornea.

**Figure 5:**
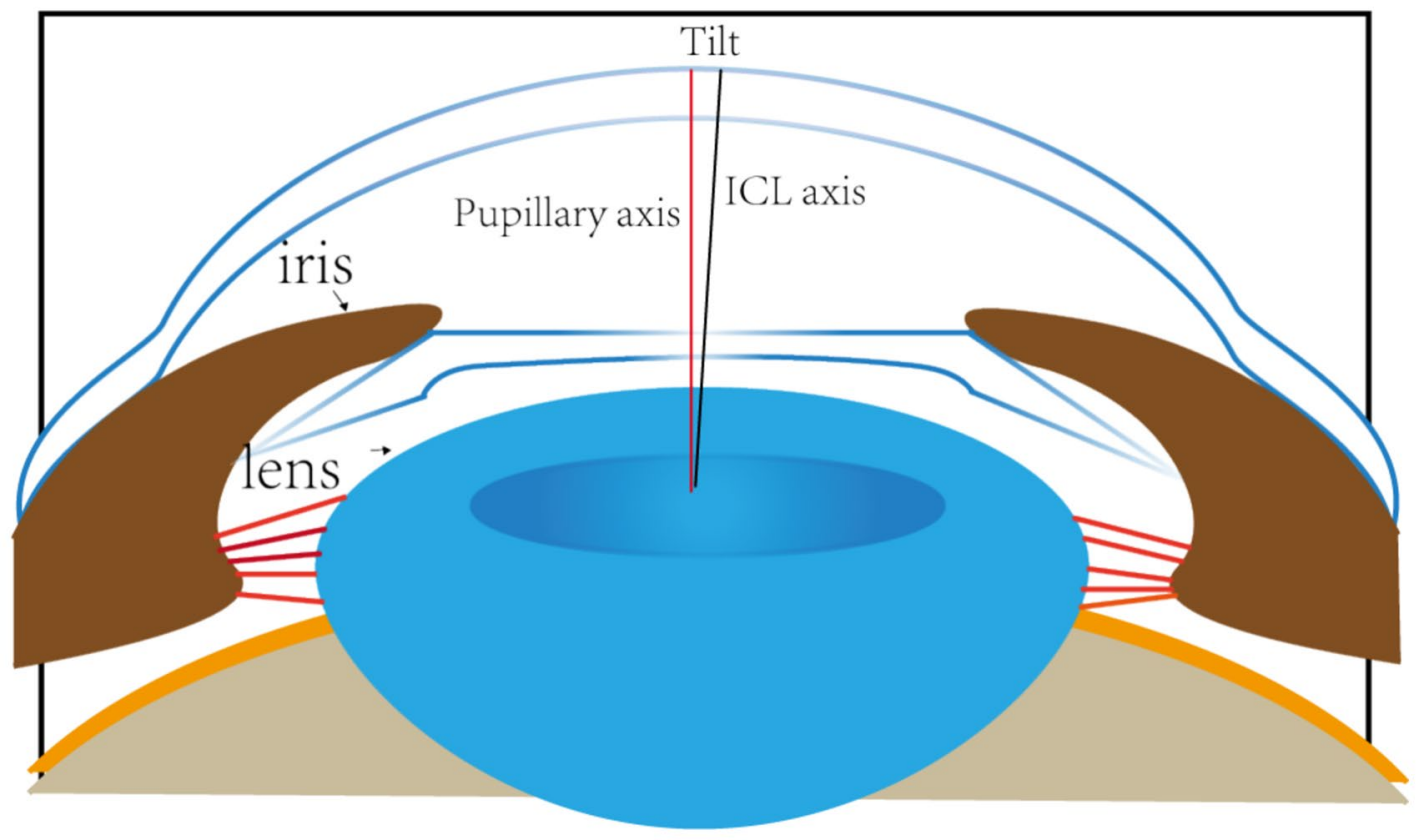
The red line represents the axis of the pupil. The black line indicates the axial position of the ICL. The angle between the two lines is the angle at which the ICL is tilted.

**Figure 6:**
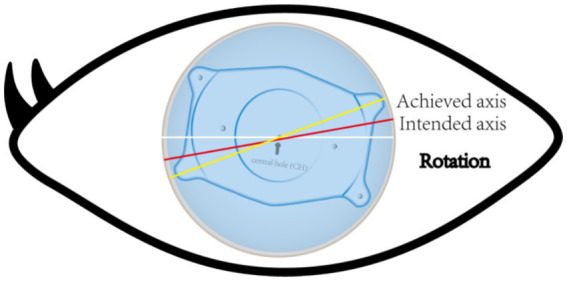
The yellow line represents the achieved axis position after ICL implantation. The red line represents the intended axis position after ICL implantation. The angle between the two is the angle rotated at a certain time after ICL implantation.

Accurate alignment of the ICL is the basis for obtaining satisfactory visual outcomes. ICL dislocation (eccentricity and tilt) is thought to contribute to HOA development and optical quality, and ICL dislocation is a major cause of a variety of postoperative complications (38–40). Although the centralization and axis alignment of a V4c ICL are important for improving optical quality after implantation, issues with alignment or axis rotation are unavoidable. The eccentricity of the ICL was detected by estimating the deviation of the center of the ICL from the center of the cornea (Figure 4), and the central hole of the ICL is usually not located in the center of the pupil. A study of ICLs in which the pupil center was used as the reference revealed that 48.9 and 93.6% of eyes were within 0.36 mm and 0.72 mm of decentration after ICL implantation, respectively (41). Niu et al. reported that 47.4% of the cases were within 0.2 mm of eccentricity, and 98.5% were within 0.5 mm. In addition, the maximum eccentricity value did not exceed 0.6 mm (31). Several studies have analyzed the associations between ICL eccentricity and ocular aberrations. Perez et al. previously compared the effects of different degrees of decentration on the development of HOAs after ICL implantation and reported that an increased number of coma aberrations was significantly associated with decentration (14). In another study, the degree of eccentricity did not affect HOA development (42). However, regardless of whether the results are relevant, all of these investigators revealed an ICL eccentricity of less than 0.5 mm, which is not sufficient to affect visual quality. Therefore, additional studies are needed to confirm the effect of ICL eccentricity on HOA induction. The ICL tilt was calculated as the angle between the pupil axis and the ICL axis (Figure 5). In a study by Niu et al., 28.1% of ICLs were within 2.0° after surgery, and 91.85% were within 4.0° after surgery, with a maximum tilt of 5.0° (31). However, the average tilt angle of the ICL in Wei et al.’s study was 2.43 ± 1.35°, which was not sufficient to cause changes in the number of HOAs (12). Similarly, the smaller the inclination angle after ICL is, the better the visual outcome. Tilt was divided into horizontal tilt and vertical tilt, but total tilt and horizontal tilt were positively correlated with the frequency and severity of vision-specific distress symptoms. Owing to the aspherical shape of the ICL optical zone, decentration and tilt have minimal effect on objective visual quality, and the effect on postoperative visual acuity is not clinically significant. In Holladay’s study, the critical values of decentration and tilt were found to be 0.4 mm and 7°, respectively, beyond which visual function was affected (43). Moreover, a significant positive correlation was noted between tilt and eccentricity. ICL rotation is the angle between the expected and actual axes of the ICL after mydriasis (Figure 6). Wei et al. reported a significant negative correlation between the postoperative vault and ICL rotation but a positive correlation with the preoperative anterior chamber depth (ACD) (12). In the study by Park et al., the rotation angle was also significantly correlated with the spherical power of the ICL (44).

### 3.2 Pupil size

On the other hand, the diameter of both scotopic and photopic pupils are reduced after ICL implantation, which also causes changes in postoperative visual quality. Pupil diameter decreases after ICL implantation, and mechanical stimulation of uveal tissue by lens implantation and surgical intervention is presumed to be responsible for this phenomenon (18). In refractive surgery, the correlation and influence of pupil diameter on postoperative visual quality (QoV) have been demonstrated (45–48). The study revealed a negative correlation between pupil diameter and daytime and nighttime QoV, indicating that a larger pupil diameter is associated with worse postoperative QoV scores (18). Zhang et al. reported that individuals with scotopic pupil diameters between 4.00 and 4.99 mm had a better mean QoV at night (18). Pupil diameter is a major factor affecting the development of HOAs. A larger pupil diameter is associated with larger aberrations and has a significant effect on retinal images. The main reasons for the degradation of retinal image quality are diffraction, aberration, and scattering. Although diffraction is clinically relevant for small pupils (3 mm), aberrations and scattering (stray light) tend to affect the QoV of pupils with larger diameters (49). Therefore, the pupil diameter is a key parameter to consider before ICL implantation. The contrast and spatial resolution of retinal images can be improved by eliminating ocular optical aberrations. This mainly depends on the pupil diameter, especially in patients with large pupil diameters at night. Therefore, the reduction in pupil diameter after ICL implantation may be beneficial to patients to some extent, as a smaller pupil diameter could help patients achieve better QoV and lower visual impairment scores after surgery. Small pupils improve visual acuity and thus discrimination of subtle stimuli, whereas large pupils increase light influx and thus detection of faint stimuli. Similarly, Chen et al. reported that patients with a smaller pupil diameter also had a smaller halo radius after ICL implantation (50).

### 3.3 Different types of ICLs

**Figure 7:**
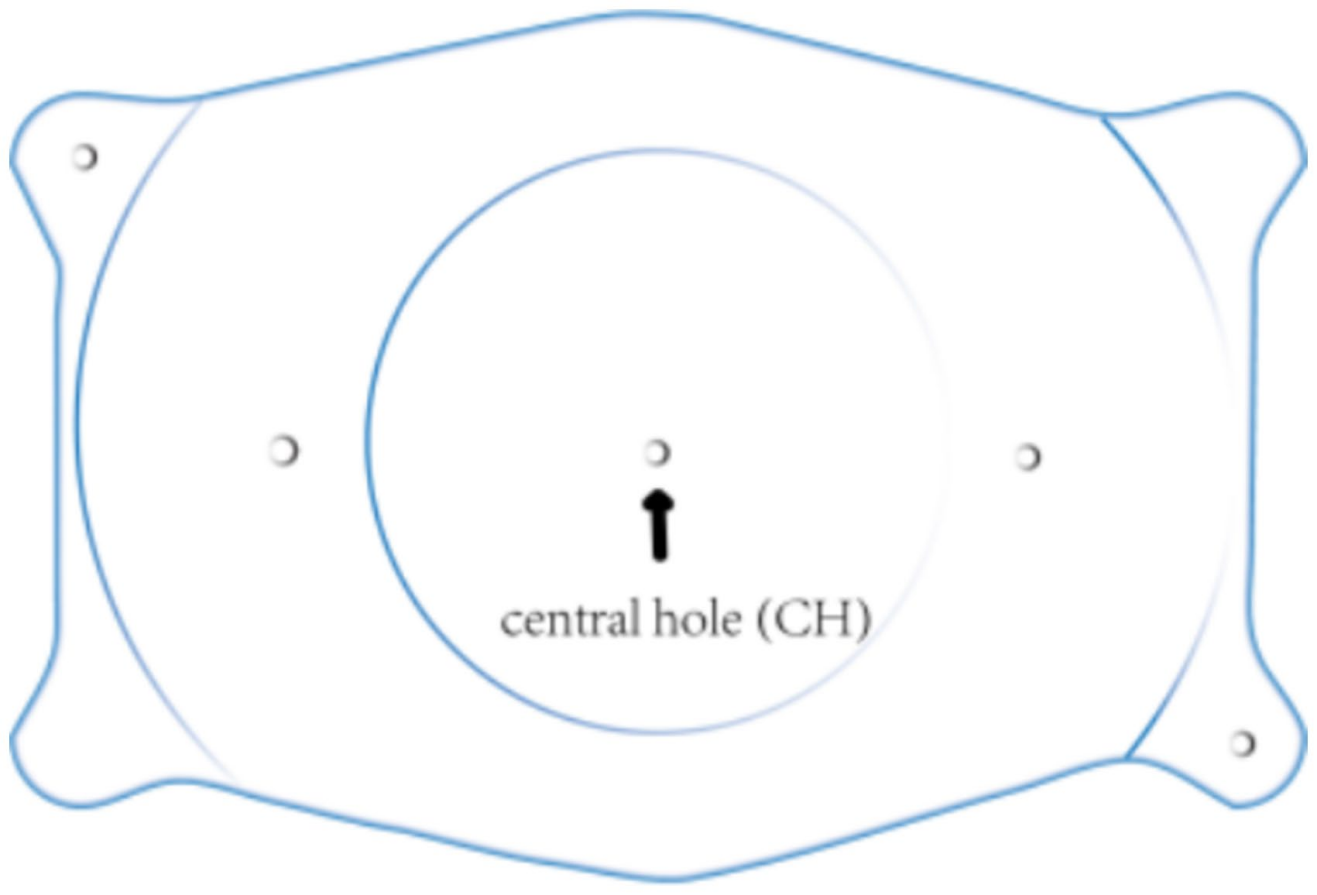
The arrow indicates the central hole in the V4c ICL model.

One of the most commonly used intraocular phasic lenses worldwide is the implantable collamer lens (ICL; STAAR Surgical, Inc., Monrovia, CA, United States) (51, 52). ICL models have been designed progressively, from the previous models (V0, V1, V2, V3, and V4) to the currently available V4b, V4c, and V5. The implantation of V4b, as well as the previous models, requires peripheral iridotomy to facilitate aqueous humor flow. On the other hand, the V4c (also known as EVO) and V5 (EVO+) models are designed with a central hole, which allows aqueous humor to circulate naturally and allows sufficient fluid to flow to maintain the normal physiology of the anterior segment, thus avoiding laser iridotomy and reducing the risks of cataract development and endothelial cell loss (Figure 7), (53, 54). Both models can be used to correct myopia and myopic astigmatism. The V4c ICL model has been widely used for the past decade. According to the results of *in vitro* and *in vivo* optical experiments, the optical quality of the V4c ICL model is good and comparable to that of the same model without the central hole (55). Both models provided good optical quality, and no significant effect on postoperative visual acuity or optical quality was noted. Two models of the ICL are available for EVO and EVO+ (29, 56, 57). The optical diameter region of the EVO model is between 4.9 and 5.8 mm. The EVO+ optical area has been expanded from 5.0 to 6.1 mm, resulting in better visual quality, especially under low light conditions. Kojima et al. reported that because the optical zone of the EVO+ model is larger, less night vision impairment is noted after implantation, and postoperative visual impairment is reduced (58). Contrast sensitivity improved after both ICL EVO and ICL EVO+ implantation, and optical quality parameters such as the modulation transfer function and scatter also improved after ICL implantation.

Toric ICLs (TICLs) also offer effective vision restoration for astigmatism sufferers. Implantable ICLs and TICLs have comparable efficacy, safety, and predictability; induce acceptable amounts of HOAs; and achieve satisfactory correction of myopia and myopic astigmatism (19). However, ICL astigmatism is a risk factor for increased halo frequency, severity, and distress effects. Some studies have shown that the incidence of halos after TICL is significantly greater than that after ICL (19). Kamiya et al. reported increases in the numbers of third-order aberrations and total HOAs in both 4-mm and 6-mm pupils 1 year after TICL implantation for the correction of moderate to high myopic astigmatism (59). However, TICL did not induce more HOAs than ICL did. One possible explanation is that the curvature gradient at the edge of the optical zone is more complex because of the astigmatism of the ICL, with different lens powers for different axes. If the TICL is too small, the vault after surgery is low and increases the possibility of IOL rotation, which reduces the astigmatism correction effect. Stabilizing the rotation of astigmatic ICLs is key to achieving efficacy. It is generally accepted that 10° rotation away from the predetermined axis after ICL implantation increases the diopter and reduces the optical performance (60). When the rotation degree reaches 30°, the astigmatism correction effect disappears (61, 62).

### 3.4 Dry eye complications

Dry eye is a common postoperative complication of refractive surgery and a multifactorial chronic ocular surface disease that is characterized by abnormal tear quality, volume and dynamics, leading to hyperosmolarity of the tear film, an imbalance in tear film homeostasis, further inflammatory responses, structural and functional damage, and abnormal ocular surface nerve sensation. Eventually, patients may experience eye discomfort, such as dryness, burning, photophobia, foreign body sensation, swelling and pain, and fluctuating vision, all of which can negatively impact their daily work, quality of life, and mental health (63–65). Compared with corneal laser surgery, ICL implantation surgery involves changing the refractive state by implanting an artificial lens without altering the ocular surface structure, thus minimally impacting tear film stability and reducing the incidence of postoperative dry eye and meibomian gland loss. The possible causes of dry eye complications after ICL implantation surgery are as follows: 1. Incision injury: Incision injury to nerve fibers leads to decreased corneal sensation and reduced levels of nutritional factors. 2. Drugs: Antibiotics and anesthetic drugs used during the perioperative period may have toxic effects. 3. Surgical stimulation: Mechanical damage during surgery and postoperative inflammatory responses can cause cell apoptosis or dysfunction, thereby reducing tear film stability. Moreover, a study by Yao et al. revealed that ICL implantation with larger and deeper incisions led to poorer postoperative tear film stability, with a greater decline than that noted after SMILE surgery and a slower recovery. The instability of the tear film causes light scattering, reducing visual quality and causing visual fatigue (66). The increase in optical aberrations may further cause various discomforts, such as blurred vision, halos, glare, and diplopia (67), which can exacerbate visual fatigue. Therefore, routine examinations of accommodative function and the non-invasive tear break-up time (NBUT) before ICL surgery are recommended to identify risk factors and design personalized treatment plans to minimize postoperative visual fatigue symptoms and optimize surgical outcomes.

## 4 Measures to improve visual quality after ICL implantation

Current measures to improve visual quality after ICL implantation include reducing the pupil size, modifying the procedure, and using relaxing corneal incisions. As mentioned above, a smaller pupil is conducive to improving the quality of vision after ICL implantation and achieving good visual outcomes. Brimonidine tartrate (0.2%) ophthalmic solution (68), which shrinks the pupil, is an effective postoperative treatment option to improve night vision quality early after ICL implantation; it also improves glare or halo symptoms in patients after LASIK under scotopic conditions (69). Chen et al. (68) revealed that the visual quality of eyes implanted with ICLs improved and reached the maximum value 1.5 h after the use of brimonidine eye drops. In addition, the pupil diameter reached the minimum value during scotopia, and the OSI value began to decrease 0.5 h after the administration of the medication and reached the minimum value at 1.5 h. Moreover, the patient’s subjective visual symptoms, such as glare or halo symptoms, decreased or even disappeared as the pupil diameter decreased. However, excessive miosis may increase patient discomfort by causing visual dimming, diffraction, and other visual disturbances due to the presence of the central foramen itself.

On the other hand, some studies have been performed to improve postoperative visual quality by altering the ICL implantation procedure. Quin et al. modified traditional ICL implantation to “pure ICL implantation” (70). The pure ICL implantation method with continuous infusion of balanced salt solution (BSS).

through a lateral incision without the use of an ophthalmic viscosurgical device (OVD) allows faster ICL implantation without contacting the lens or corneal endothelium and without the need to rinse the OVD, thus shortening the operation time, reducing the cost of the material, and improving patients’ and surgeons’ experiences. Moreover, the quality of vision in the early period after pure ICL implantation is reported to be better than that after conventional implantation, as evidenced by 1-day postoperative MTF and SR values and 1-week postoperative OV100%, OV20%, and OV9% values that were greater than those after conventional implantation (70). Wang et al. also modified the double-incision ophthalmic viscosurgical device (OVD)-free approach to improve visual quality after ICL implantation (71). The incidence of ring-shaped dysphotopsia was significantly lower in the no-OVD group than in the standard group, and the severity of this symptom and the level of distress were significantly lower in the no-OVD group. Wang et al. suggested that OVD retention in the foramen may cause stray light, which induces ring-shaped dysphotopsia, and that the disappearance of this symptom is accompanied by OVD absorption (71). Uncorrected distance visual acuity (UDVA) and corrected distance visual acuity (CDVA) were also greater in the simple group compared with the conventional ICL implantation group on the first postoperative day, as ICL implantation alone facilitated the control of early postoperative intraocular pressure (IOP) and the anterior chamber inflammatory reaction (ACR). At 3 months after ICL implantation, good visual quality was observed. Liu et al. studied surgical incisions to minimize preexisting low-grade astigmatism (up to 1.0 D) as well as surgically induced astigmatism affecting postoperative visual quality (72). A steep meridian corneal release incision (SM-CRI) was found to reduce corneal astigmatism by flattening the steep meridian and steepening the flat meridian through a corneal release incision. Moreover, this simple, cost-effective procedure has been widely used in cataract surgery to correct low to moderate corneal astigmatism. Hei et al. reported that patients with an SM-CRI had a significantly lower incidence of postoperative corneal astigmatism, a significantly lower incidence of postoperative irregular astigmatism, and better postoperative visual quality than patients with a non-steep meridional corneal release incision (NSM-CRI) did (73). The advantage of an SM-CRI over other types of incisions is that an SM-CRI in ICL implantation avoids TICL misalignment, thereby reducing the risk and cost of ICL placement complications.

## 5 Conclusion

The negative effects of myopia, especially high myopia, on patients’ visual function, quality of life, and productivity have been well described. For patients with high myopia or a thin cornea, implantable lenses (ICLs) may represent an alternative to corneal refractive surgery (CRS). Implantable collagen lens (ICL) implantation, a safe, effective, predictable and stable refractive surgery, has received extensive attention in recent years. ICLs are removable and can correct a wide range of myopia (−0.50D to −18.00D), which is suitable for patients with high myopia, moderate myopia, mild myopia and myopic astigmatism. Owing to its unique advantages, the ICL can also be used for the treatment of vision regression after corneal refractive error correction, when good postoperative visual quality has also been achieved. ICLs can effectively improve visual acuity in patients with super high myopia, especially young patients. ICLs may completely correct refractive errors and therefore improve patients’ quality of life and reduce the burden on public health. Although most patients are satisfied or highly satisfied with the outcome of ICL implantation, subjective symptoms such as halos and glare remain sources of concern. These symptoms may affect patients’ visual quality and quality of life. Methods to improve visual quality after ICL implantation are the subject of current research. Studies have focused on improving visual quality by optimizing surgical techniques, selecting appropriate ICL models, and providing postoperative treatment.

Owing to advancements in science and technology, new ICLs are being developed. These new models may have better optical properties, a lower risk of complications, and wider applicability. Although the tilt and decentration of the ICL after implantation are within acceptable limits, their effects on visual quality are still the subject of intense research. Research to explore measures to localize the ICL more accurately and to reduce its potential impact on visual quality is ongoing. Before ICL surgery, anterior segment (AS) OCT can guide diagnostic assessment of the anterior segment and help select the correct ICL model. Moreover, new techniques to further improve the safety and effectiveness of surgery will be the focus of future research. During the ICL implantation procedure, intraoperative optical coherence tomography (iOCT) can help evaluate the qualitative and quantitative impacts of surgical intervention on tissues, provide real-time dynamic feedback, and offer better parameters for intraoperative and postoperative management. These new techniques may include methods to achieve more accurate surgical positioning, methods to improve surgical efficiency, and methods to reduce surgical complications. Although ICL implantation has been shown to be safe and effective in the short term, its long-term effects still need to be further evaluated. Future studies should focus on long-term visual quality and complications after ICL implantation, as well as patient satisfaction with surgical outcomes. In the future, ICL implantation may be integrated with other ophthalmic technologies, such as corneal cross-linking technology and laser surgery, to further improve surgical outcomes and patients’ visual quality.

In conclusion, ICL implantation, as a safe and effective refractive surgical procedure, has broad application prospects in the correction of myopia and myopic astigmatism. However, methods to improve the safety and effectiveness of surgery, enhance postoperative visual quality and reduce the risk of complications are topics of current research. Future research will focus on personalized treatment, the development of new surgical techniques, the evaluation of long-term results, and integration with other technologies.

## References

[1] Holden BA Fricke TR Wilson DA Jong M Naidoo KS Sankaridurg P. Global Prevalence of Myopia and High Myopia and Temporal Trends from 2000 Through 2050. Ophthalmology. 123:1036–42. doi: 10.1016/j.ophtha.2016.01.006 26875007

[2] Packer M. The implantable Collamer Lens with a central port: Review of the literature. Clin Ophthalmol (Auckland, NZ). (2018) 12:2427–38. doi: 10.2147/OPTH.S188785 PMC6267497 30568421

[3] Luo W Aruma A Li M Wang J Xie J Xiao X. Four-year visual outcomes and optical quality of SMILE and implantable collamer lens V4c (EVO-ICL) implantation for high myopia: a retrospective study. BMC Ophthalmol. (2023) 23:341. doi: 10.1186/s12886-023-03050-9, PMID: 37525155 PMC10392000

[4] Li K Wang Z Wang MX. Implantable collamer lens implantation (ICL) versus small incision lenticule extraction (SMILE) in low to moderate myopia: study protocol for a randomized, non-inferiority trial. Trials. (2022) 23:910. doi: 10.1186/s13063-022-06851-3, PMID: 36307873 PMC9617386

[5] Zhang J He F Liu Y Fan X. Implantable collamer lens with a central hole for residual refractive error correction after corneal refractive surgery. Exp Ther Med. (2020) 20:160. doi: 10.3892/etm.2020.9289, PMID: 33093898 PMC7571336

[6] Jiang Z Wang H Luo D-Q Chen J. Optical and visual quality comparison of implantable collamer lens and femtosecond laser assisted laser *in situ* keratomileusis for high myopia correction. Int J Ophthalmol. (2021) 14:737–43. doi: 10.18240/ijo.2021.05.15, PMID: 34012890 PMC8077018

[7] Qin Q Wu Z Bao L Chen H Yang L He Z. Evaluation of visual quality after EVO-ICL implantation for hypermyopia an observational study. Medicine. (2019) 98:e17677. doi: 10.1097/MD.0000000000017677, PMID: 31689784 PMC6946539

[8] Wei R Li M Zhang H Aruma A Miao H Wang X. Comparison of objective and subjective visual quality early after implantable collamer lens V4c (ICL V4c) and small incision lenticule extraction (SMILE) for high myopia correction. Acta Ophthalmol. (2020) 98:E943–50. doi: 10.1111/aos.14459, PMID: 32419383

[9] Niu L Miao H Tian M Fu D Wang X Zhou X. One-year visual outcomes and optical quality of femtosecond laser small incision lenticule extraction and Visian implantable Collamer Lens (ICL V4c) implantation for high myopia. Acta Ophthalmol. (2020) 98:E662–7. doi: 10.1111/aos.14344, PMID: 32003129

[10] Tian H Gao W Xu C Wang Y. Clinical outcomes and higher order aberrations of wavefront-guided LASIK versus SMILE for correction of myopia: a systemic review and meta-analysis. Acta Ophthalmol. (2023) 101:606–18. doi: 10.1111/aos.15638, PMID: 36726315

[11] Montes-Mico R Pastor-Pascual F Artiaga-Elordi E Ruiz-Mesa R Tana-Rivero P. *In vivo* optical quality of posterior-chamber phakic implantable collamer lenses with a central port. Eye Vision. (2021) 8:30. doi: 10.1186/s40662-021-00251-5, PMID: 34392836 PMC8365931

[12] Wei P-H Li J Jiao XI Yu Z Song H. Short-term clinic observation of misalignment and rotational stability after implantable collamer lens implantation. Graefes Arch Clin Exp Ophthalmol. (2023) 261:1473–81. doi: 10.1007/s00417-022-05929-7 36484805

[13] Perez-Vives C Dominguez-Vicent A Ferrer-Blasco T Pons AM Montes-Mico R. Optical quality of the Visian implantable Collamer Lens for different refractive powers. Graefes Arch Clin Exp Ophthalmol. (2013) 251:1423–9. doi: 10.1007/s00417-012-2200-8, PMID: 23142994

[14] Perez-Vives C Ferrer-Blasco T Madrid-Costa D Garcia-Lazaro S Montes-Mico R. Optical quality comparison of conventional and hole-Visian implantable col lamer Lens at different degrees of decentering. Am J Ophthalmol. (2013) 156:69–76.e1. doi: 10.1016/j.ajo.2013.01.030, PMID: 23540712

[15] Kayhan B Coskunseven E Sahin O Pallikaris I. The effects of implantable collamer lens implantation on higher order aberrations. Int J Ophthalmol. (2019) 12:1848–52. doi: 10.18240/ijo.2019.12.05, PMID: 31850167 PMC6901894

[16] Chen H Liu Y Feng X Niu G Fan Y. Long-term clinical observation of posterior chamber Phakic intraocular Lens implantation in young population. Eye Contact Lens. (2018) 44:S365–9. doi: 10.1097/ICL.0000000000000497, PMID: 29944497

[17] Wang Y Zhao KX Jin Y Niu YF Zuo T. Changes of higher order aberration with various pupil sizes in the myopic eye. J Refract Surg. (2003) 19:S270–4. doi: 10.3928/1081-597X-20030302-21, PMID: 12699188

[18] Zhang Q Wu Y Huang H Qin G Li L Chen J. The influence of pupil diameter upon and subjective quality of vision following implantable collamer lens (ICL V4c) implantation an observational study. Medicine. (2023) 102:e35198. doi: 10.1097/MD.0000000000035198, PMID: 37800803 PMC10553097

[19] Wei R Li M Niu L Aruma A Miao H Shen Y. Comparison of visual outcomes after non-toric and toric implantable collamer lens V4c for myopia and astigmatism. Acta Ophthalmol. (2021) 99:511–8. doi: 10.1111/aos.14652, PMID: 33084228

[20] Cao K Zhang J Wang J Yusufu M Jin S Chen S. Implantable collamer lens versus small incision lenticule extraction for high myopia correction: a systematic review and meta-analysis. BMC Ophthalmol. (2021) 21:450. doi: 10.1186/s12886-021-02206-9, PMID: 34961514 PMC8711178

[21] Shimizu K Kamiya K Igarashi A Shiratani T. Early clinical outcomes of implantation of posterior chamber phakic intraocular lens with a central hole (hole ICL) for moderate to high myopia. Br J Ophthalmol. (2012) 96:409–12. doi: 10.1136/bjophthalmol-2011-300148, PMID: 21733922

[22] Igarashi A Kamiya K Shimizu K Komatsu M. Visual performance after implantable Collamer Lens implantation and Wavefront-guided laser in situ Keratomileusis for high myopia. Am J Ophthalmol. (2009) 148:164–170.e1. doi: 10.1016/j.ajo.2009.02.001, PMID: 19375059

[23] Fu M Li M Xian Y Yu Z Zhang H Choi J. Two-year visual outcomes of evolution implantable Collamer Lens and small incision Lenticule extraction for the correction of low myopia. Front Med. (2022) 9:780000. doi: 10.3389/fmed.2022.780000, PMID: 35492322 PMC9043127

[24] Shin JY Ahn H Seo KY Kim EK Kim T-I. Comparison of higher order aberrations after implantable Collamer Lens implantation and Wavefront-guided LASEK in high myopia. J Refract Surg. (2012) 28:106–11. doi: 10.3928/1081597X-20111018-02, PMID: 22074464

[25] Miao H He L Shen Y Li M Yu Y Zhou X. Optical quality and intraocular scattering after femtosecond laser small incision Lenticule extraction. J Refract Surg. (2014) 30:296-+. doi: 10.3928/1081597X-20140415-02, PMID: 24893354

[26] Chen T Yu F Lin H Zhao Y Chang P Lin L. Objective and subjective visual quality after implantation of all optic zone diffractive multifocal intraocular lenses: a prospective, case-control observational study. Br J Ophthalmol. (2016) 100:1530–5. doi: 10.1136/bjophthalmol-2015-307135, PMID: 26903522

[27] Kamiya K Shimizu K Igarashi A Kobashi H. Effect of femtosecond laser setting on visual performance after small-incision lenticule extraction for myopia. Br J Ophthalmol. (2015) 99:1381–7. doi: 10.1136/bjophthalmol-2015-306717, PMID: 25855501

[28] Qin Q Bao L Yang L He Z Huang Z. Comparison of visual quality after EVO-ICL implantation and SMILE to select the appropriate surgical method for high myopia. BMC Ophthalmol. (2019) 19:21. doi: 10.1186/s12886-019-1029-x, PMID: 30732575 PMC6367781

[29] Martinez-Plaza E Lopez-Miguel A Lopez-de la Rosa A McAlinden C Fernandez I Maldonado MJ. Effect of the EVO plus Visian Phakic implantable Collamer Lens on visual performance and quality of vision and life. Am J Ophthalmol. (2021) 226:117–25. doi: 10.1016/j.ajo.2021.02.005, PMID: 33577790

[30] He T Zhu Y Zhou J. Optical quality after posterior chamber Phakic implantation of an intraocular Lens with a central hole (V4c implantable Collamer Lens) under different lighting conditions. BMC Ophthalmol. (2020) 20:82. doi: 10.1186/s12886-020-01340-0, PMID: 32131800 PMC7055093

[31] Niu L Zhang Z Miao H Zhao J Li M He JC. Effects of tilt and decentration of Visian implantable Collamer Lens (ICL V4c) on visual quality: an observational study. BMC Ophthalmol. (2022) 22:294. doi: 10.1186/s12886-022-02499-4, PMID: 35790941 PMC9254425

[32] Zhan B Huang Y Chen X Aruma A Cheng M Wang X. Comparison of long-term visual quality after keratorefractive lenticule extraction and implantable collamer lens V4c for high myopia. J Cataract Refract Surg. (2024) 50:1157–64. doi: 10.1097/j.jcrs.0000000000001523, PMID: 39025652

[33] Eom Y Kim DW Ryu D Kim J-H Yang SK Song JS. Ring-shaped dysphotopsia associated with posterior chamber phakic implantable collamer lenses with a central hole. Acta Ophthalmol. (2017) 95:E170–8. doi: 10.1111/aos.13248, PMID: 27678470

[34] Liu T Linghu S Pan L Shi R. Effects of V4c-ICL implantation on myopic Patients' vision-related daily activities. J Ophthalmol. (2016) 2016:1–6. doi: 10.1155/2016/5717932, PMID: 27965890 PMC5124673

[35] Mohr N Dirisamer M Siedlecki J Mayer WJ Schworm B Harrant L. Determinants of subjective quality of vision after Phakic intraocular Lens implantation. J Refract Surg. (2022) 38:280-+. doi: 10.3928/1081597X-20220405-01, PMID: 35536709

[36] Siedlecki J Schmelter V Mayer WJ Schworm B Priglinger SG Dirisamer M. SMILE versus implantable Collamer Lens implantation for high myopia: a matched comparative study. J Refract Surg. (2020) 36:150-+. doi: 10.3928/1081597X-20200210-02, PMID: 32159819

[37] Eppig T Spira C Tsintarakis T El-Husseiny M Cayless A Mueller M. Ghost-image analysis in phakic intraocular lenses with central hole as a potential cause of dysphotopsia. J Cataract Refract Surg. (2015) 41:2552–9. doi: 10.1016/j.jcrs.2015.05.034, PMID: 26703506

[38] Ashena Z Maqsood S Ahmed SN Nanavaty MA. Effect of intraocular Lens tilt and Decentration on visual acuity, Dysphotopsia and Wavefront aberrations. Vision (Basel, Switzerland). (2020) 4:41. doi: 10.3390/vision4030041 32937750 PMC7559075

[39] Liu X Xie L Huang Y. Effects of decentration and tilt at different orientations on the optical performance of a rotationally asymmetric multifocal intraocular lens. J Cataract Refract Surg. (2019) 45:507–14. doi: 10.1016/j.jcrs.2018.10.045, PMID: 30947854

[40] Shi M Kong J Li X Yan Q Zhang J. Observing implantable collamer lens dislocation by panoramic ultrasound biomicroscopy. Eye. (2015) 29:499–504. doi: 10.1038/eye.2014.336, PMID: 25613840 PMC4816352

[41] Park MJ Jeon HM Lee KH Han SY. Comparison of postoperative optical quality according to the degree of decentering of V4c implantable collamer lens. Int J Ophthalmol. (2017) 10:619–23. doi: 10.18240/ijo.2017.04.19 28503437 PMC5406642

[42] Miao A Zhang M Chen T Lu Y. The influence of Decentration on higher-order aberrations in artisan Aphakic intraocular Lens implantation eyes. J Ophthalmol. (2020) 2020:1–7. doi: 10.1155/2020/7601524, PMID: 32351725 PMC7171672

[43] Holladay JT Piers PA Koranyi G van der Mooren M Norrby NES. A new intraocular lens design to reduce spherical aberration of pseudophakic eyes. J Refract Surg. (2002) 18:683–91. doi: 10.3928/1081-597X-20021101-04, PMID: 12458861

[44] Park SC Kwun YK Chung E-S Ahn K Chung T-Y. Postoperative astigmatism and Axis stability after implantation of the STAAR Toric implantable Collamer Lens. J Refract Surg. (2009) 25:403–9. doi: 10.3928/1081597X-20090422-01, PMID: 19507791

[45] Liu Q Yang X Lin L Liu M Lin H Liu F. Review on centration, astigmatic Axis alignment, pupil size and optical zone in SMILE. Asia-Pacific J Ophthalmol. (2019) 8:385–90. doi: 10.1097/01.APO.0000580144.22353.46, PMID: 31567265 PMC6784779

[46] Myung D Schallhorn S Manche EE. Pupil size and LASIK: a review. J Refract Surg. (2013) 29:734-+. doi: 10.3928/1081597X-20131021-02, PMID: 24203804

[47] Ratra V Lam DSC. Small pupil--big problem: a management algorithm. Asia-Pacific J Ophthalmol (Philadelphia, PA). (2015) 4:131–3. doi: 10.1097/APO.0000000000000119 26065497

[48] Pazo EE McNeely RN Richoz O Nesbit MA Moore TCB Moore JE. Pupil influence on the quality of vision in rotationally asymmetric multifocal IOLs with surface-embedded near segment. J Cataract Refract Surg. (2017) 43:1420–9. doi: 10.1016/j.jcrs.2017.08.013, PMID: 29223231

[49] Xu R Wang H Thibos LN Bradley A. Interaction of aberrations, diffraction, and quantal fluctuations determine the impact of pupil size on visual quality. J Opt Soc Am Opt Image Sci Vis. (2017) 34:481–92. doi: 10.1364/JOSAA.34.000481, PMID: 28375317

[50] Chen X Han T Zhao F Miao H Wang X Zhou X. Evaluation of disk halo size after implantation of a Collamer Lens with a central hole (ICL V4c). J Ophthalmol. (2019) 2019:1–6. doi: 10.1155/2019/7174913, PMID: 31485347 PMC6710753

[51] Igarashi A Shimizu K Kamiya K. Eight-year follow-up of posterior chamber Phakic intraocular Lens implantation for moderate to high myopia. Am J Ophthalmol. (2014) 157:532–539.e1. doi: 10.1016/j.ajo.2013.11.006, PMID: 24239774

[52] Jiménez-Alfaro I del Castillo JMB García-Feijoó J de Bernabé JGG de la Iglesia JMS. Safety of posterior chamber phakic intraocular lenses for the correction of high myopia -: anterior segment changes after posterior chamber phakic intraocular lens implantation. Ophthalmology. (2001) 108:90–9. doi: 10.1016/S0161-6420(00)00403-6, PMID: 11150270

[53] Alfonso JF Lisa C Fernandez-Vega L Almanzar D Perez-Vives C Montes-Mico R. Prevalence of cataract after collagen copolymer phakic intraocular lens implantation for myopia, hyperopia, and astigmatism. J Cataract Refract Surg. (2015) 41:800–5. doi: 10.1016/j.jcrs.2014.07.039, PMID: 25840304

[54] Goukon H Kamiya K Shimizu K Igarashi A. Comparison of corneal endothelial cell density and morphology after posterior chamber phakic intraocular lens implantation with and without a central hole. Br J Ophthalmol. (2017) 101:1461–5. doi: 10.1136/bjophthalmol-2016-309363, PMID: 28292776

[55] Uozato H Shimizu K Kawamorita T Ohmoto F. Modulation transfer function of intraocular collamer lens with a central artificial hole. Graefes Arch Clin Exp Ophthalmol. (2011) 249:1081–5. doi: 10.1007/s00417-010-1602-8, PMID: 21229257

[56] Martinez-Plaza E Lopez-de la Rosa A Lopez-Miguel A Holgueras A Maldonado MJ. EVO/EVO plus Visian implantable Collamer lenses for the correction of myopia and myopia with astigmatism. Expert Rev Med Devices. (2023) 20:75–83. doi: 10.1080/17434440.2023.2174429, PMID: 36708714

[57] Packer M Alfonso JF Aramberri J Elies D Fernandez J Mertens E. Performance and safety of the extended depth of focus implantable Collamer® Lens (EDOF ICL) in Phakic subjects with presbyopia. Clin Ophthalmol. (2020) 14:2717–30. doi: 10.2147/OPTH.S271858, PMID: 32982164 PMC7509320

[58] Kojima T Kitazawa Y Nakamura T Takahashi M Kamiya K Ichikawa K. Prospective randomized multicenter comparison of the clinical outcomes of V4c and V5 implantable Collamer lenses: a contralateral eye study. J Ophthalmol. (2018) 2018:1–6. doi: 10.1155/2018/7623829, PMID: 30254757 PMC6145048

[59] Kamiya K Shimizu K Aizawa D Igarashi A Komatsu M Nakamura A. One-year follow-up of posterior chamber Toric Phakic intraocular Lens implantation for moderate to high myopic astigmatism. Ophthalmology. (2010) 117:2287–94. doi: 10.1016/j.ophtha.2010.03.054 20598749

[60] Kamiya K Shimizu K Kobashi H Igarashi A Komatsu M. Three-year follow-up of posterior chamber Toric Phakic intraocular Lens implantation for moderate to high myopic astigmatism. PLoS One. (2013) 8:e56453. doi: 10.1371/journal.pone.0056453, PMID: 23409187 PMC3568037

[61] Zhu M Zhu L Zhu Q Xu C Yu P Xiao H. Clinical effect and rotational stability of TICL in the treatment of myopic astigmatism. J Ophthalmol. (2020) 2020:1–7. doi: 10.1155/2020/3095302, PMID: 33489326 PMC7803223

[62] Langenbucher A Viestenz A Szentmary N Behrens-Baumann W Viestenz A. Toric intraocular lenses-theory, matrix calculations, and clinical practice. J Refract Surg. (2009) 25:611–22. doi: 10.3928/1081597X-20090610-07, PMID: 19662918

[63] Bitar MS Olson DJ Li M Davis RM. The correlation between dry eyes, anxiety and depression: the sicca, anxiety and depression study. Cornea. (2019) 38:684–9. doi: 10.1097/ICO.0000000000001932, PMID: 30950896

[64] Magno MS Utheim TP Snieder H Hammond CJ Vehof J. The relationship between dry eye and sleep quality. Ocul Surf. (2021) 20:13–9. doi: 10.1016/j.jtos.2020.12.009, PMID: 33421635

[65] Sayegh RR Yu Y Farrar JT Kuklinski EJ Shtein RM Asbell PA. Ocular discomfort and quality of life among patients in the dry eye assessment and management study. Cornea. (2021) 40:869–76. doi: 10.1097/ICO.0000000000002580 33290317 PMC8175479

[66] Yao J Feng J Li W Liu C Li Y Wang X. SMILE and ICL implantation on the ocular surface and meibomian glands in patients with postoperative myopia. BMC Ophthalmol. (2024) 24:522. doi: 10.1186/s12886-024-03790-2, PMID: 39633296 PMC11619467

[67] Liu H-T Zhou Z Luo W-Q He W-J Agbedia O Wang J-X. Comparison of optical quality after implantable collamer lens implantation and wavefront-guided laser *in situ* keratomileusis. Int J Ophthalmol. (2018) 11:656–61. doi: 10.18240/ijo.2018.04.20, PMID: 29675387 PMC5902373

[68] Chen X Wang X Naidu RK Qian Y Miao H Zhou X. Effect of brimonidine tartrate 0.2% ophthalmic solution on visual quality after implantable collamer lens implantation with a central hole. Int Ophthalmol. (2020) 41:293–301. doi: 10.1007/s10792-020-01581-4, PMID: 33175316

[69] Lee JH You YS Choe CM Lee ES. Efficacy of brimonidine tartrate 0.2% ophthalmic solution in reducing halos after laser in situ keratomileusis. J Cataract Refract Surg. (2008) 34:963–7. doi: 10.1016/j.jcrs.2008.01.028, PMID: 18499002

[70] Qin Q Bao L He Z Chen F Zhu D Zhang S. Pure ICL implantation: a novel ophthalmic Viscosurgical device-free method. J Ophthalmol. (2021) 2021:1–11. doi: 10.1155/2021/7363267, PMID: 34659826 PMC8514915

[71] Wang C Yu Q Zhou Q Li F Zhou J. Clinical outcomes of a modified ophthalmic viscosurgical device-free implantable collamer lens implantation. Indian J Ophthalmol. (2024) 72:1291–7. doi: 10.4103/IJO.IJO_2859_23, PMID: 38767536 PMC11552826

[72] Liu S Liu J Lin F Yu L Cheng C Wang T. Efficacy comparison between steep-Meridian incision and non-steep-Meridian incision in implantable Collamer Lens surgery with low-to-moderate astigmatism. Ophthalmol Therapy. (2023) 12:1711–22. doi: 10.1007/s40123-023-00704-1, PMID: 37016057 PMC10164207

[73] He W Zhu X Du Y Yang J Lu Y. Clinical efficacy of implantation of toric intraocular lenses with different incision positions: a comparative study of steep-axis incision and non-steep-axis incision. BMC Ophthalmol. (2017) 17:132. doi: 10.1186/s12886-017-0528-x, PMID: 28750611 PMC5531017

